# Stress-induced hyperglycemia and expression of glucose cell transport genes in skeletal muscle of critically ill patients: a cross-sectional study

**DOI:** 10.20945/2359-4292-2024-0417

**Published:** 2025-03-27

**Authors:** Priscila Bellaver, Daisy Crispim, Lílian Rodrigues Henrique, Cristiane Bauermann Leitão, Ariell Freires Schaeffer, Tatiana Helena Rech, Diego Paluszkiewicz Dullius

**Affiliations:** 1 Programa de Pós-Graduação em Ciências Médicas: Endocrinologia, Universidade Federal do Rio Grande do Sul, Porto Alegre, RS, Brasil; 2 Unidade de Terapia Intensiva, Hospital de Clínicas de Porto Alegre, Porto Alegre, RS, Brasil; 3 Faculdade de Medicina, Universidade Federal do Rio Grande do Sul, Porto Alegre, RS, Brasil; 4 Divisão de Cirurgia Plástica, Hospital de Clínicas de Porto Alegre, Porto Alegre, RS, Brasil; 5 Grupo de Diabetes e Metabolismo, Centro de Pesquisa Clínica e Divisão Endocrinologia, Hospital de Clínicas de Porto Alegre, Porto Alegre, RS, Brasil; 6 Departamento de Medicina Interna, Universidade Federal do Rio Grande do Sul, Porto Alegre, RS, Brasil

**Keywords:** Diabetes mellitus, Glycemic control, Insulin receptor substrate proteins, Glucose transporter type 4

## Abstract

**Objective:**

To explore the association between diabetes and stress-induced hyperglycemia with skeletal muscle expression of key genes related to glucose transport.

**Methods:**

This is a cross-sectional study. Skeletal muscle biopsies were taken from the left vastus muscle of critically ill adult patients within 24 hours of intensive care unit admission, and the expression of the genes of interest, namely insulin receptor substrate 1 (IRS1), insulin receptor substrate 2 (IRS2), solute carrier family 2 member 1 (SLC2A1), and solute carrier family 2 member 4 (SLC2A4), was analyzed using quantitative polymerase chain reaction. The primary analysis was planned to compare the gene expression pattern between patients with and without diabetes mellitus. The secondary analyses compared the gene expression in subgroups of patients with different levels of glycemia, glycemic variability, and glycemic gap.

**Results:**

A total of 50 consecutive patients (15 with diabetes mellitus and 35 without diabetes mellitus) were included from April 2018 to September 2018. No differences in gene expression were found between patients with or without diabetes mellitus. Individuals with hyperglycemia > 200 mg/dL at intensive care unit admission exhibited a downregulation of IRS1 compared to those without (0.4 [0.1-0.8] *versus* 1.1 [0.3-2.2], p = 0.04). Similarly, patients with a glycemic gap ≥ 80 mg/dL exhibited a downregulation of IRS1 compared to those with a glycemic gap < 80 mg/dL (0.3 [0.1-0.7] *versus* 1 [0.4-2] p=0.04). There was no difference in gene expression between patients with glycemic variability higher or lower than 40 mg/dL.

**Conclusion:**

No significant changes were found in skeletal muscle expression of IRS1, IRS2, SLC2A1, and SLC2A4 in critically ill patients with or without diabetes mellitus. However, IRS1 was downregulated in patients with stress-induced hyperglycemia.

## INTRODUCTION

Diabetes mellitus (DM) imposes a substantial disease burden on outpatients, but it is not associated with higher mortality rates in the critical illness setting, a phenomenon known as the diabetes paradox (^1,2^). However, stress-induced hyperglycemia, glycemic variability, and glycemic gap are associated with unfavorable outcomes, especially in individuals without DM (^2,3^). The rationale for this protective influence of pre-existing DM during stress-induced hyperglycemia is not entirely clear (^3^). Some authors have suggested that chronic hyperglycemia may generate a protective cellular conditioning against damage mediated by acute stress-induced hyperglycemia (^2^). This cellular conditioning mechanism would consist of the downregulation of the glucose transporter-1 (GLUT-1) and the glucose transporter-4 (GLUT-4) due to chronic exposure to hyperglycemia.

The family 2 member 1 solute-carrier gene (*SLC2A1*) encodes GLUT-1, which is widely distributed throughout tissues. GLUT-1 holds a high transport capacity and high affinity for the glucose molecule in an insulin-independent manner (^4,5^). GLUT-4, encoded by the solute-carrying gene of family 2 member 4 (*SLC2A4*), is regulated by insulin and expressed abundantly in skeletal muscle and in adipose tissue (^4,6^). In the absence of insulin, this membrane protein is restricted within muscle and adipose cytoplasm. Upon insulin stimulation, vesicles containing this protein on their surface are translocated and incorporated to the plasma cell membrane, allowing glucose transport into the cell (^4,7^).

The translocation of GLUT-4 to the cell membrane is orchestrated by a cascade of metabolic reactions (^7^). Briefly, upon insulin binding to its cell surface receptor, tyrosine kinase is activated. This activation leads to the generation of second messenger proteins, specifically insulin receptor substrate 1 (IRS1) and insulin receptor substrate 2 (IRS2). IRS1 associates with the enzyme phosphatidylinositol 3-kinase, which is essential for GLUT-4 translocation (^7^). However, in critically ill patients, GLUT-4 faces a potential reduction due to multiple factors (^8^). Notably, catecholamine and glucocorticoid levels reduce tyrosine kinase activity, whereas inflammatory cytokines, especially tumor necrosis factor, act by reducing tyrosine phosphorylation, subsequently downregulating IRS1 (^9^).

Patients exposed to chronic hyperglycemia seem to have reduced insulin signaling in skeletal muscle. We recently described the downregulation of *INSR* in the skeletal muscle of critically ill patients with stress-induced hyperglycemia. In order to better understand the phenomena, we aim to explore the association between diabetes and stress-induced hyperglycemia with skeletal muscle expression of key genes related to glucose transport.

## METHODS

This is a single center cross-sectional study. The study protocol was approved by the Research Ethics Committee at the *Hospital de Clínicas de Porto Alegre* (project No. 2017-0386). Informed consent was obtained from the patients or their legal representatives. The study strictly adheres to the principles set forth in the Helsinki Declaration and follows the determinations of Brazilian Resolution 466/2012 of the National Health Council (^10^).

### Study population

The study population was recruited from April 2018 to September 2018 and consisted of adult individuals (aged over 18) who were admitted to the intensive care unit (ICU). Patients with diabetic ketoacidosis, hyperosmolar hyperglycemic state, hemoglobinopathies, pregnancy, and coagulopathies (platelet count < 100,000/uL or use of anticoagulants) were excluded.

### Data collection

Clinical and laboratory data were obtained prospectively from electronic medical records and previously described in detail (^11^). Blood samples for serum blood glucose and glycated hemoglobin (HbA1c) level quantifications were taken from all patients at study entry.

Diabetes was defined on the basis of a prior diagnosis assessed by electronic chart review or when HbA1c ≥ 6.5% (^12^). Hyperglycemia was defined according to the threshold proposed by the American Diabetes Association (ADA) for in-hospital hyperglycemia as any blood glucose measurement > 140 mg/dL (^12^). Severe hyperglycemia was defined as blood glucose > 200 mg/dL (^13^). Glucose variability was calculated as the absolute difference in capillary blood glucose levels within the initial 24 hours at the ICU. Variability was categorized as low or high based on a cutoff value of 40 mg/dL (^13^). The glycemic gap was calculated as the difference between serum blood glucose upon ICU admission and the estimated mean blood glucose derived from HbA1c values, as previously described (^13^). The glycemic gap was categorized as low or high based on a cutoff value of 80 mg/dL (^13^). The Simplified Acute Physiology Score 3 (SAPS 3) was used to score disease severity, with higher scores indicating greater severity (^14^).

### RNA isolation and quantitative real-time polymerase chain reaction

Muscle biopsies of the left vastus lateral muscle were performed as previously described within the first 24 hours of ICU admission (^11^). Briefly, the specimens were isolated, washed in ice-cold 50 mM phosphate buffer saline, gently dried, immersed in RNALater solution (Thermo Fisher Scientific, Waltham, Massachusetts, USA), and stored at -80 °C until gene expression analyses.

The relative quantification of the mRNA of the genes of interest was carried out using the quantitative real-time polymerase chain reaction (RT-qPCR) method. Total RNA were extracted from skeletal muscle tissue cells using the PureLink RNA Mini Kit (Thermo Fisher Scientific). The concentration and quality of the RNA samples were analyzed using a NanoDrop One spectrophotometer (Thermo Fisher Scientific). Reverse transcription of 200 ng of RNA into complementary DNA (cDNA) was carried out using the SuperScript™ IV VILO™ (Thermo Fisher Scientific), following the manufacturer’s protocol.

The cDNA was then amplified by qPCR, which was carried out by monitoring the increase in fluorescence of the SYBR Green dye in real time. Specific primers for *IRS1, IRS2, SLC2A1*, and *SLC2A4* were designed using published human sequences and Primer Express 3.0 software (Thermo Fisher Scientific). Supplementary Table 1 contains the sequence information for all primers.

All qPCR reactions were performed in a ViiA 7 RT-PCR System (Thermo Fisher Scientific). Each qPCR reaction contained 5 µL of 1X PowerUp SYBR Green Master Mix 1x;Thermo Fisher Scientific), 0.5 µL (1 ng/µL) of forward and reverse specific primers, 1 µL of cDNA (200 ng), and sterile water to complete 10 µL. Samples were analyzed in triplicates and a negative control was included in each qPCR plate. The relative expression of each gene was performed by relative quantification using the comparative ∆∆C_q_ method (^15^) and using peptidyl-prolyl isomerase A (*cyclophilin A* - *PPIA*) as the reference gene. The validation assays were carried out by amplifying the targets and reference genes separately using serial dilutions of a mix of cDNA samples. As a requirement of this method, the target and reference genes exhibited equal amplification efficiencies. The ∆∆C_q_ method calculates changes in gene expression as relative n-fold changes between an experimental and an external calibrator sample (^15,16^). Quantitative qPCR specificity was determined using melting curve analyses, and all primers generated amplicons that produced a single, sharp peak during the analyses.

### Statistical analysis

A sample size of 50 patients was estimated to detect a 30% reduction in the expression of genes related to glucose transport in patients with or without DM, with a power of 80% and a significance level of 0.05 based on the study of Schefold et al., which found a downregulation of *SLC2A4* in critically ill patients with polyneuropathy (^17^). The primary analysis planned was to compare the pattern of gene expression between patients with and without DM. Secondary analyses compared gene expression in subgroups of patients with severe hyperglycemia (cutoff 200 mg/dL), glycemic variability (cutoff 40 mg/dL), and glycemic gap (cutoff 80 mg/dL).

Categorical variables were expressed as percentages. Variables with normal distribution are presented as mean ± standard deviation. Variables with skewed distribution were log-transformed before analysis and are presented as median and interquartile intervals. Groups were compared using the Student’s *t*-test, or Chi-squared test, as appropriate. Statistical analyses were performed in the Statistical Package for the Social Sciences, version 21.0 (Chicago, IL, USA) software program.

## RESULTS

### Patient characteristics

From April 2018 to September 2018, a cohort of 204 patients admitted to the ICU was screened for eligibility and 50 patients were included. Supplementary Figure 1 shows the screening and reasons for exclusions.

**Figure 1:**
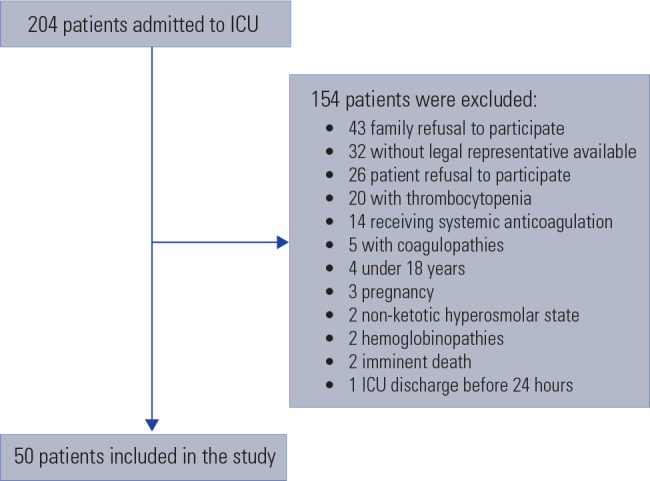
Screening and reasons for exclusions.

Out the patients, 31 (59%) were male, with a mean age of 63 ± 15 years. Acute respiratory failure was the reason for ICU admission in 15 patients (30%). The mean SAPS 3 score was 63 ± 17. Fifteen patients had a pre-existing diagnosis of DM (30%). The subgroup of patients with and without DM generally had similar baseline characteristics, except for glucose control. As expected, patients with DM had higher blood glucose (287 ± 186 *versus* 96 ± 51 mg/dL; p=0.001), HbA1c values (6.9 ± 2.3 *versus* 5.7 ± 0.6%; p = 0.001), and received higher insulin doses than those without DM (47% *versus* 14%; p < 0.001). The overall mortality rate was 46%, similar between the two groups (53% in patients with DM *versus* 43% in patients without DM; p = 0.19).

### Primary analysis

Table 1 shows the skeletal muscle expression of *IRS1, IRS2, SLC2A1*, and *SLC2A4* in patients with and without DM. No significant difference was identified between patients with or without DM regarding the expression of these genes.

**Table 1:** Gene expression in skeletal muscle biopsies from patients with or without diabetes mellitus

Gene	Diabetes mellitus (n = 15)	Without diabetes mellitus (n = 35)	p-value
SLC2A1	0.7 (0.3-1.5)	0.6 (0.35-1.2)	0.9
SLC2A4	0.94 (0.55-2.1)	1.04 (0.5-1.6)	0.9
IRS1	0.6 (0.4-1.8)	0.9 (0.3-2)	0.7
IRS2	0.94 (0.8-1.15)	1 (0.6-1.8)	0.6

Results are expressed in n-fold changes (∆∆Cq method) and described as median and interquartile range.

### Secondary analysis

Table 2 shows the association between glycemic parameters and the expression of *IRS1, IRS2, SLC2A1*, and *SLC2A4* in skeletal muscle. Patients with hyperglycemia above 200 mg/dL showed a downregulation of *IRS1* compared to those below this cutoff. There was no difference in gene expression between patients with glycemic variability below or above 40 mg/dL.

**Table 2:** Association between gene expression and hyperglycemia, glycemic variability, and stress-induced hyperglycemia

Gene	Hyperglycemia			Glycemic variability			Glycemic gap		
	No (n = 30)	Yes (n = 16)	p-value	< 40 mg/dL (n = 36)	≥ 40 mg/dL (n = 14)	p-value	< 80 mg/dL (n = 8)	≥ 80 mg/dL (n = 42)	p-value
SLC2A1	0.6 (0.4-1.3)	0.7 (0.3-1.4)	0.9	0.5 (0.5-0.9)	0.7 (0.3-1.5)	0.9	0.6 (0.3-1.3)	0.7 (0.4-1.4)	0.9
SLC2A4	1.1 (0.5-1.7)	0.8 (0.6-1.9)	0.7	1.3 (0.2-2.2)	1 (0.6-1.6)	0.7	1 (0.5-1.7)	0.9 (0.5-1.9)	0.8
IRS1	1.1 (0.3-2.2)	0.4 (0.1-0.8)	0.04	1.7 (0.4-2)	0.6 (0.3-1.9)	0.3	0.96 (0.4-2)	0.3 (0.1-0.7)	0.04
IRS2	1 (0.65-1.9)	1 (0.8-1.3)	0.5	1.55 (0.5-2.1)	1 (0.7-1.6)	0.6	0.9 (0.6-1.7)	1 (0.8-1.3)	0.6

Hyperglycemia was defined as any serum glucose > 200 mg/dL. Glycemic variability was calculated as the absolute difference in capillary blood glucose during the first intensive care unit day. Glycemic gap was calculated by the difference between the serum glucose at intensive care unit admission and the estimated mean blood glucose derived from glycated hemoglobin. The values are expressed in changes n times (∆∆Cq method) and described as median and interquartile range.

ICU: intensive care unit.

To separate the effects of a chronically altered metabolic state from those attributed to acute stress-induced hyperglycemia, the glycemic gap was assessed. The expression of *IRS1* was downregulated in patients with a glycemic gap above 80 mg/dL. No significant differences were observed in the expression of *IRS2, SLC2A1*, and *SLC2A4* based on the glycemic gap cutoff of 80 mg/dL.

### Exploratory analysis

Skeletal muscle expression of *IRS1, IRS2, SLC2A1*, and *SLC2A4* was similar between patients who received insulin therapy and those who did not (Supplementary Table 2).

## DISCUSSION

In this observational study including 50 critically ill patients, no early differences were identified in the expression of *IRS1, IRS2, SLC2A1*, and *SLC2A4* between patients with or without DM. However, it is noteworthy that *IRS1*, a key gene related to cellular glucose metabolism, was downregulated in patients with stress-induced hyperglycemia.

The interplay between gene expression and glucose metabolism in critically ill patients is complex. Although gene expression patterns may provide insights into its mechanisms, stress-induced hyperglycemia is multifactorial. Many elements, such as the individual patient characteristics, the nature and severity of critical illness and its treatments, and the specific genes and pathways involved, might collectively contribute to its occurrence. The *IRS1*, identified in this study as downregulated in the context of stress-induced hyperglycemia, plays a key role in cellular glucose transport and insulin signaling (^4^). Recently, our group also demonstrated a downregulation of *INSR* in critically ill patients (^11^). However, it is essential to recognize the intricate involvement of a series of cofactors in this cascade, making the attribution of a single factor as the culprit for the phenomena challenging (^7,17^). Studies have shown that changes in microRNAs are associated with insulin resistance, suggesting a crucial role of epigenetics in modulating the metabolic response (^18,19^).

Altered glucose parameters are associated with unfavorable clinical outcomes in critically ill patients (^13,20^). We analyzed the *IRS1, IRS2, SLC2A1*, and *SLC2A4* gene expressions and their association with glycemic parameters. Previous studies suggest that prior exposure to hyperglycemia might mitigate the impact of glycemic variability on mortality of critically ill patients with DM (^21^). We identified *IRS1* as differentially expressed between patients with or without acute hyperglycemia, but gene expression was not influenced by glycemic variability.

The glycemic gap separates the impact of stress-induced hyperglycemia from chronic hyperglycemia. In our sample, patients with DM had a significantly higher glycemic gap compared to those without DM, demonstrating the influence of acute and chronic hyperglycemia on these individuals. Our findings showed that patients with a higher glycemic gap have a significant downregulation of the *IRS1* gene. Studies have suggested that polymorphisms in the *IRS1* gene could be biomarkers of insulin resistance in healthy individuals and might play a role in acute hyperglycemia among patients with DM (^22^). This becomes particularly interesting because *IRS1* is downregulated by inflammatory cytokines and might therefore be associated with the severity of critical illness (^23,24^), which in turn is associated with the severity of acute hyperglycemia (^25^).

This is the first study to evaluate the role of skeletal muscle gene expression of glucose transporters and insulin signaling genes in critically ill patients with stress-induced hyperglycemia, with or without diabetes. However, it holds some limitations. Firstly, the lack of assessment of the effect of epigenetic factors on gene regulation limits its conclusions. Indeed, exploring DNA methylation patterns would be a further step. Secondly, this study evaluated a restricted set of genes. Therefore, studies including other genes related to glucose metabolism are necessary. Thirdly, only early biopsies were performed, precluding the detection of late changes in genes influenced by critical illness-induced inflammation. Sequential biopsies would provide valuable insights. Fourthly, the information on calorie consumption and insulin doses was not described due to inaccuracy of data, therefore preventing conclusions about their potential influence on gene expression.

In conclusion, no significant changes were found in skeletal muscle gene expression of *IRS1, IRS2, SLC2A1*, and *SLC2A4* in critically ill patients with or without DM, but interestingly, stress-induced hyperglycemia downregulated *IRS1*. Research focused on gene expression represents a valuable contribution to elucidate the impact of certain genes and pathways on the outcomes of critically ill patients with DM. However, our study is one piece of the intricate puzzle: does this genetic difference simply add one more association between glucose metrics and severity of illness, or help to define a causal relationship where the gene itself worsens the glycemic metric and may thereby worsen outcomes? Further research exploring a broader molecular landscape, including epigenetic modifications and gene expression alterations induced by inflammation and hyperglycemia overtime, is needed to comprehensively unravel the genetic and molecular aspects contributing to stress-induced hyperglycemia in critical illness.

## SUPPLEMENTARY

**Supplementary Table 1:** Oligonucleotide primers used for quantitative real-time polymerase chain reaction

IRS1	5’ AAGAAGTGGCGCCACAAGTC 3’
	5’ CAGCCCGCTTGTTGATGTT 3’
IRS2	5’ GCAGAACATCCACGAGACCAT 3’
	5’ GGAACTCGAAGAGCTCCTTGAG 3’
SLC2A1	5’ CGGGCCAAGAGTGTGCTAA 3’
	5’ TCCTTCATCTCCRGCAGGTCAT 3’
SLC2A4	5’ TTCATCATTGGCATGGGTTTC 3’
	5’ GGAGGACCGCAAATAGAAGGA 3’

**Supplementary Table 2:** Gene expression in skeletal muscle tissue from survivors and non-survivors and in patients who received and did not receive insulin therapy

Gene	Insulin therapy (n = 11)	No insulin therapy (n = 39)	p-value
SLC2A1	0.96 (0.29-1.4)	0.6 (0.35-1.4)	0.9
SLC2A4	1.2 (0.6-3.02)	0.95 (0.05-4.2)	0.3
IRS1	0.4 (0.2-1.1)	0.9 (0.3-2.03)	0.23
IRS2	0.99 (0.8-1.3)	0.97 (0.6-1.7)	0.6

Results expressed in changes n times (∆∆Cq method) and described as median and interquartile range.
